# Targeting γ-aminobutyric acid type A receptors to mitigate perioperative neurocognitive disorders: a narrative review

**DOI:** 10.1016/j.bja.2026.01.004

**Published:** 2026-04-02

**Authors:** Joycelyn Ba, Connor T.A. Brenna, Lilia Kaustov, Dian-Shi Wang, Beverley A. Orser

**Affiliations:** 1 Temerty Faculty of Medicine, University of Toronto, Toronto, ON, Canada; 2 Perioperative Brain Health Centre, Sunnybrook Health Sciences Centre, Toronto, ON, Canada; 3 Department of Physiology, University of Toronto, Toronto, ON, Canada; 4 Department of Anesthesiology & Pain Medicine, University of Toronto, Toronto, ON, Canada; 5 Department of Anesthesiology and Pain Medicine, Sunnybrook Health Sciences Centre, Toronto, ON, Canada

**Keywords:** α5 subunit, anaesthetic drugs, δ subunit, GABA_A_ receptors, neuroinflammation, perioperative neurocognitive disorders, postoperative delirium

## Abstract

Perioperative neurocognitive disorders are common complications after anaesthesia and surgery, particularly among older adults. A growing body of preclinical evidence implicates dysregulation of extrasynaptic γ-aminobutyric acid type A (GABA_A_) receptors as a key contributor to these disorders. Unlike synaptic GABA_A_ receptors that mediate synaptic or phasic inhibition, extrasynaptic GABA_A_ receptors, that often contain α5 and δ receptor subunits, generate a tonic inhibitory conductance that regulates neuronal excitability and network dynamics. Most anaesthetic drugs are well known to potentiate fast GABAergic inhibitory neurotransmission, but these agents also trigger persistent increases in the cell-surface expression of extrasynaptic GABA_A_ receptors, especially in brain regions that are critical for cognition such as the hippocampus and prefrontal cortex. Surgery and inflammation similarly cause excess cell-surface expression of extrasynaptic GABA_A_ receptors. The increased number of receptors enhances the amplitude of a tonic inhibitory current, which disrupts network plasticity and impairs learning, memory, and executive function. This narrative review explores the mechanistic links between perioperative care and alterations in the cell-surface expression of extrasynaptic GABA_A_ receptors, and the implications of such mechanisms in perioperative neurocognitive disorders. On the basis of these insights, we propose several potential prevention and treatment strategies.

**Editor's key points**

• Preclinical evidence suggests that excess extrasynaptic GABA_A_ receptor function contributes to perioperative neurocognitive disorders.
• Numerous general anaesthetic drugs, as well as inflammation following surgery, could contribute to this dysregulation.
• Maintaining normal extrasynaptic GABA_A_ receptor function after surgery is a promising therapeutic strategy to prevent and treat these disorders.

Perioperative neurocognitive disorders (PNDs) are among the most prevalent and debilitating postoperative complications, impacting up to 40% of older patients undergoing surgery.^1^ These conditions, which encompass postoperative delirium, delayed neurocognitive recovery, and postoperative neurocognitive disorders, are associated with increased mortality, healthcare costs, institutionalisation, and long-term cognitive decline.^2^^,^^3^

Despite the high incidence of PNDs, the lack of a clear aetiology has hampered the development of effective prevention and treatment strategies. Competing hypotheses that have emerged from preclinical studies have focused on imbalances in inhibitory and excitatory neurotransmission, mitochondrial dysfunction, oxidative stress, disruption of the blood–brain barrier with an influx of immune cells, and activation of glia.^4^, ^5^, ^6^, ^7^, ^8^, ^9^, ^10^ Identifying which of these are plausible biological targets to mitigate PNDs remains a research priority.

Increasing evidence suggests that dysregulation of extrasynaptic γ-aminobutyric acid type A (GABA_A_) receptors contributes to postoperative cognitive deficits.^11^ Our laboratory first identified excess cell-surface expression of GABA_A_ receptors in neurones as a potential causal factor in studies aimed at understanding how general anaesthetic drugs cause long-term cognitive deficits after the drugs have been eliminated from the body.^12^ Subsequently, it was observed that drugs in other classes including the non-opioid analgesic gabapentin, and pathophysiological processes such as inflammation and surgery, similarly increase cell-surface expression of GABA_A_ receptors in neurones.^13^, ^14^, ^15^

This narrative review summarises the key preclinical studies that support the "excess cell-surface GABA_A_ receptor" hypothesis of PNDs, in which aberrant overexpression of receptors on the surface of neurones contributes to cognitive dysfunction (Fig. 1). We then propose that reducing cell-surface expression or activity of GABA_A_ receptors is a plausible therapeutic strategy to spare cognition, and identify four approaches including (1) repurposing agents such as dexmedetomidine and ketamine to prevent excess cell-surface expression of GABA_A_ receptors, (2) using negative allosteric modulators (NAMs) to directly inhibit excess extrasynaptic GABA_A_ receptor activity, (3) disrupting the anchoring of extrasynaptic GABA_A_ receptors to the cytoskeleton with peptide-based therapeutics, and (4) avoiding gabapentin in high-risk patient populations. These approaches offer promise to preserve brain function after anaesthesia and surgery. Also, given that excess GABA_A_ receptor function contributes to other disorders including stroke, traumatic brain injury, and neurodegenerative disease,^16^^,^^17^ determining whether targeting GABA_A_ receptors preserves cognition in surgical patients will have implications that extend well beyond the perioperative environment.

**Fig 1:**
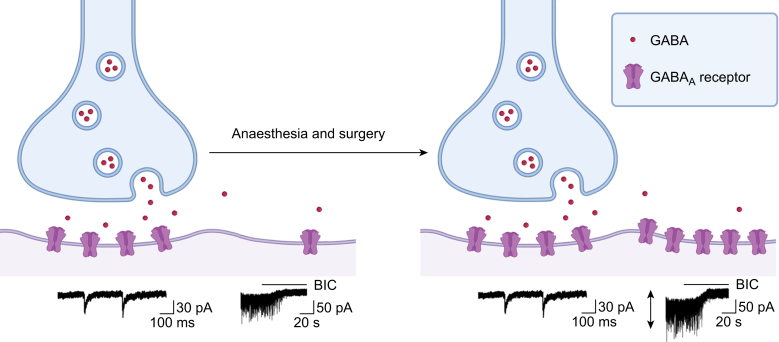
The excess cell-surface γ-aminobutyric acid type A receptor (GABA_A_) receptor hypothesis of perioperative neurocognitive deficits. Perioperative factors, including most general anaesthetic drugs, surgery, and inflammation, are proposed to increase the cell-surface expression of extrasynaptic GABA_A_ receptors in the hippocampus and other brain regions. This increase in receptor number and function increases the amplitude of a tonic inhibitory conductance revealed by application of bicuculine (BIC; a competitive antagonist of the GABA_A_ receptor), which leads to cognitive deficits. Created in BioRender.

## Overview of γ-aminobutyric acid type A receptors

GABA_A_ receptors are the most abundant inhibitory receptors in the mammalian brain. These pentameric transmitter-activated ion channels assemble from 19 different subunits (ɑ1–6, β1–3, γ1–3, δ, ε, π, θ, and ρ1–3).^18^ Most GABA_A_ receptors are composed of two ɑ subunits, two β subunits, and one γ or δ subunit, which are arranged around a central ion channel pore.^16^^,^^18^^,^^19^ Binding of GABA causes a series of conformational changes that open the channel pore, allowing negatively charged ions (typically chloride) to move down an electrochemical gradient across the cell membrane. Channel opening and the influx of anions hyperpolarise the cell membrane and decrease membrane resistance causing shunting of excitatory current, which dampens the impact of depolarisation and reduces neuronal firing.^20^

Most GABA_A_ receptors form clusters in synaptic or extrasynaptic regions of neurones.^21^ Synaptic GABA_A_ receptors are activated by high, transient concentrations of GABA, which is released from presynaptic terminals.^22^ The vesicular release of GABA from presynaptic terminals activates clusters of postsynaptic GABA_A_ receptors, which generate fast, transient or ‘phasic’, postsynaptic currents.^22^ In contrast, extrasynaptic GABA_A_ receptors are localised beyond synapses. For example, they are enriched along the somatodendritic membrane of pyramidal neurones or on axons at sites far from synapses.^23^^,^^24^ These extrasynaptic receptors are activated by low ambient levels of GABA that spill over from synapses, including those of neighbouring neurones, or GABA that is released via non-vesicular sources including GABA cotransporters or from anion channels in astrocytes.^12^^,^^22^^,^^25^ These receptors generate a sustained, low-amplitude, ‘tonic’ inhibitory conductance that reduces neuronal firing and controls network oscillations, synaptic plasticity, and cognitive processes.^12^^,^^25^ Most extrasynaptic GABA_A_ receptors contain either α5 subunits (α5GABA_A_ receptors) or a δ subunit (δGABA_A_ receptors).^16^^,^^22^

It is well known that both synaptic and extrasynaptic GABA_A_ receptors are primary targets of many important neurodepressive drugs including volatile or inhaled anaesthetics, propofol, etomidate, neurosteroids, barbiturates, and benzodiazepines.^26^^,^^27^ At clinically relevant concentrations, these drugs act as positive allosteric modulators (PAMs) of GABA_A_ receptors.^27^ The resulting increase in channel opening typically prolongs the decay of synaptic currents, increases the amplitude of the tonic inhibitory current, or both.^26^^,^^28^ Ketamine and nitrous oxide are better known as antagonists of the *N*-methyl-D-aspartate (NMDA) subtype of glutamate receptor.^29^ However, nitrous oxide also acts as a PAM of GABA_A_ receptors,^30^ whereas ketamine allosterically potentiates the function of extrasynaptic, but not synaptic, GABA_A_ receptors.^31^

Changes in GABA_A_ receptor activity resulting from allosteric modulators are widely recognised;^32^ however, alterations in cell-surface expression that robustly modify the efficacy of GABAergic inhibition are not widely appreciated. The number of GABA_A_ receptors on the plasma membrane is not static, but rather is determined by a balance between the trafficking of receptors to the plasma membrane (exocytosis) and the internalisation of receptors (endocytosis).^33^ Multiple factors regulate exocytosis and endocytosis, including the subunit composition of GABA_A_ receptors, the phosphorylation of the subunits, and interactions with key anchoring proteins that link receptors to cytosolic scaffolding proteins.^33^ Importantly, anaesthetic drugs and a variety of additional factors trigger rapid and sustained changes in cell-surface expression of GABA_A_ receptors.^34^ As outlined in this review, a new and transformative era of GABA_A_ receptor pharmacology is emerging that focuses on modifying cell-surface expression of GABA_A_ receptors as a therapeutic strategy. Several of these strategies are potentially relevant to mitigating PNDs.

## Extrasynaptic γ-aminobutyric acid type A receptors

Among the various subtypes of extrasynaptic GABA_A_ receptors, α5GABA_A_ receptors have been the most strongly implicated in causing cognitive deficits after anaesthesia and surgery in animal models.^14^^,^^34^ These receptors are highly expressed in the hippocampus, a region involved in learning and memory, as well as the olfactory bulb, with lower levels present in the cortex, amygdala, thalamus, hypothalamus, and midbrain.^35^ ɑ5GABA_A_ receptors account for only 5% of all GABA_A_ receptors in the brain; however, they constitute 25% of GABA_A_ receptors in the hippocampus.^35^^,^^36^ The kinetic and pharmacological properties of α5GABA_A_ receptors as well as their patterns of expression are highly conserved in rodents and humans, and are distinct from those of other GABA_A_ receptor subpopulations.^37^^,^^38^ Several lines of evidence indicate that α5GABA_A_ receptors critically regulate hippocampal-dependent memory processes.^34^

The activity of extrasynaptic δGABA_A_ receptors is also of interest in surgical patients. These receptors are expressed in the cerebellum, thalamus, dorsal hippocampus, and cortex, where they generate a tonic inhibitory current.^39^ These receptors do not typically form clusters, but rather are diffusely expressed in extrasynaptic regions of neurones.^40^ In addition to allosteric upregulation in response to multiple anaesthetic drugs, δGABA_A_ receptor expression levels are sensitive to stress,^41^ ethanol, and neurosteroids,^42^ but insensitive to benzodiazepines.^20^ These unique features of δGABA_A_ receptors have contributed to the first US Food and Drug Administration-approved treatments for postpartum depression, which are neuroactive steroids that counteract changes in δGABA_A_ receptor cell-surface expression associated with mood dysregulation occuring during the postpartum period.^43^ Thus, δGABA_A_ receptors have already been shown to be an effective therapeutic target.

## Anaesthetic drugs promote surface expression of extrasynaptic γ-aminobutyric acid type A receptors

As reviewed previously, the causes of PNDs are multifactorial,^44^ and the degree to which general anaesthetic drugs contribute to these disorders in surgical patients, as compared with other causal factors including inflammation, remains uncertain.^45^^,^^46^ However, preclinical studies have clearly demonstrated that exposure to anaesthetic drugs causes subtle, detrimental effects on cognition that persist after the drugs are eliminated. A recent scoping review of preclinical studies that examined the impact of anaesthetic drugs on cognition^47^ found that the majority demonstrated post-anaesthetic cognitive deficits. Impairments were more frequent in studies of aged animal models, in studies of volatile (as compared with i.v.) anaesthetics, and after longer durations of anaesthetic drug treatment.^47^

One potential mechanism underlying deficits in memory and executive function caused by both i.v. and inhaled anaesthetic drugs is an increase in cell-surface expression of extrasynaptic GABA_A_ receptors and a resulting increase in the tonic inhibitory current.^34^ Most studies that support this proposed mechanism have identified changes in ɑ5GABA_A_ receptors after treatment with general anaesthetic drugs, and this evidence is summarised below.

### Etomidate

Pioneering studies that aimed to identify the molecular basis of persistent anaesthetic-induced alterations in ɑ5GABA_A_ receptors used etomidate, because this drug has a relatively high affinity for GABA_A_ receptors, a rapid onset of action, a short half-life, and is eliminated without producing active metabolites.^48^ Etomidate has very high specificity for GABA_A_ receptors, particularly those containing the β2 or β3 subunit, and it causes few cardiorespiratory adverse effects that confound studies of cognition.^48^

Etomidate triggers excess cell-surface expression of ɑ5GABA_A_ receptors in the hippocampus^34^^,^^49^ and increases tonic inhibitory currents measured from principal neurones in *ex vivo* hippocampal slices, when studied for up to 1 week after drug treatment.^34^^,^^49^ In contrast, etomidate causes few changes in synaptic currents that are sustained after it is eliminated.^34^ The increased tonic inhibitory current triggered by etomidate impairs long-term potentiation (LTP), a cellular correlate of memory, through ɑ5GABA_A_ receptor-dependent mechanisms.^34^ Cognitive deficits produced by etomidate in these models, particularly in hippocampus-dependent learning and memory paradigms, are similarly sustained.^34^ These deficits are likely dose dependent: a sedative etomidate dose (8 mg kg^−1^) impairs recognition memory for up to 72 h, whereas an anaesthetising dose (20 mg kg^−1^) causes subtle deficits that persist for up to 1 week.^34^ Pharmacologically inhibiting or genetically deleting ɑ5GABA_A_ receptors prevents the sustained post-anaesthetic cognitive effects caused by etomidate.^34^

### Propofol

Although numerous studies have reported that propofol acutely increases tonic current,^50^, ^51^, ^52^ impairs LTP induction,^53^ and causes sustained memory deficits in animal models,^47^ relatively few studies have examined whether clinically relevant doses of propofol cause sustained changes in extrasynaptic GABA_A_ receptor function. In one study, a single i.p. dose of propofol (100 mg kg^−1^) increased the cell-surface expression of ɑ5GABA_A_ receptors in hippocampal neurones of mice for at least 5 days.^54^ Consistent with these findings, cell-surface expression of β3 subunit-containing GABA_A_ receptor, which can include ɑ5 subunits, increases as rapidly as 5 min after drug exposure, likely attributable to reduced receptor endocytosis.^55^ To the best of our knowledge, no behavioural studies have specifically examined the impact of ɑ5GABA_A_ receptor inhibitors on propofol-induced behavioural deficits, or whether such deficits occur in genetically modified mice lacking the *Gabra5* gene that encodes the ɑ5 subunit (*Gabra*5-/- mice). Thus, the contribution of GABA_A_ receptors to memory deficits observed after a clinically relevant dose of propofol remains uncertain.

### Volatile anaesthetic drugs

Animal studies that examined the effects of volatile anaesthetic drugs on cell-surface expression of extrasynaptic ɑ5GABA_A_ receptors have reported results that vary depending on the drug dose, the age of the animals, and the time interval between drug treatment and behavioural studies.

Isoflurane administered at anaesthetising concentrations (1.3–1.4 vol%) for 1 h *in vivo* increases both the cell-surface expression of ɑ5GABA_A_ receptors and the tonic current for at least 24 h.^34^ These effects are associated with memory deficits that are prevented by L-655,708, a NAM of ɑ5GABA_A_ receptors.^56^^,^^57^ However, these effects might be sensitive to the interval between treatment and testing, and to the duration of isoflurane administration. In another study, isoflurane (1.3 vol%, administered over an unspecified duration) did not induce a sustained change in ɑ5GABA_A_ receptor cell-surface expression (at an unspecified timepoint) or alterations in LTP, when measured 7 days after treatment, or cause deficits in memory behaviours 8–11 days after drug treatment.^15^ Similarly, a brief treatment for 20 min with a sedative dose of isoflurane (0.7%) did not alter ɑ5GABA_A_ receptor cell-surface expression.^34^

Sevoflurane (3.3–4.0 vol% for 2 h), like isoflurane, increases ɑ5GABA_A_ receptor cell-surface expression, when measured at timepoints between 24 h and 7 days after treatment.^58^^,^^59^ Sevoflurane similarly increases tonic current measured 24 h and 48 h after treatment.^59^^,^^60^ Deficits in recognition and spatial memory were observed in four studies, at several timepoints up to 1 week after sevoflurane treatment.^58^^,^^59^^,^^61^^,^^62^ One study reported that sevoflurane (3.3 vol% for 2 h) increased colocalisation of ɑ5GABA_A_ receptors with radixin (its key extrasynaptic binding protein), increased the tonic current, and reduced the time course of synaptic decay, when measured 24 h after treatment,^59^ indicating alterations to both synaptic and extrasynaptic inhibitory function. Interestingly, another study has reported that sevoflurane (2.7–3.0 vol% for 1 h) decreased extrasynaptic populations of ɑ5GABA_A_ receptors.^62^ These conflicting findings likely result from differences in drug dosing and methodology as the duration of sevoflurane exposure (2 *vs* 1 h), age of the mice (18 *vs* 3 months), sex, and the assays used to assess the expression of extrasynaptic ɑ5GABA_A_ receptors (western immunoblot *vs* immunofluorescence) differed (Table 1). Age-dependent sensitivity to volatile anaesthetics may play an important role in the observed inconsistencies in outcome. One study reported contrasting results in younger and aged mice, where only aged mice were vulnerable to sevoflurane-induced increases in ɑ5GABA_A_ receptor cell-surface expression,^58^ although this result was not reported by other laboratories. Notably, in one study, older animals were observed to exhibit lower baseline levels of ɑ5GABA_A_ receptors,^63^ and thus may experience a larger relative increase, which may contribute to the increased risk of post-anaesthetic cognitive deficits. The mechanisms that account for the differences between experimental conditions, such as cell signalling systems that regulate endocytosis and exocytosis or phosphorylation states of the receptors, have not been clearly identified.

**Table 1:** Summary of preclinical *in vivo* studies investigating the effects of general anaesthetics on ɑ5 subunit mRNA and ɑ5GABA_A_ receptors protein expression. INH, inhaled; N/A, not applicable; NR, not reported.

Study (year)	Intervention				Model				Outcomes			
	General anaesthetic	Dose	Route	Duration of treatment	Species	Strain	Age	Sex	Timing between treatment and measurement	mRNA	Surface protein	Total protein
Zurek (2014)^34^	Etomidate	8 mg kg^−1^	i.p.	N/A	Mouse	C57BL/6J×SvEv129	1–4 mo	Male	24 h, 1 wk, 2 wk	NR	Increased at 24 h and 1 wk, returned to baseline by 2 wk	Unchanged at all timepoints
		20 mg kg^−1^	i.p.	N/A	Mouse	C57BL/6J×SvEv129	1–4 mo	Male	24 h	NR	Increased	Unchanged
Wang (2018)^49^	Etomidate	20 mg kg^−1^	i.p.	N/A	Mouse	C57BL/6	8–9 wk	Male	24 h	NR	Etomidate increased cell-surface expression, co-treatment with dexmedetomidine (25 μg kg^−1^, i.p.) prevented this increase	Unchanged by etomidate alone or cotreatment with dexmedetomidine (25 μg kg^−1^, i.p.)
Nagarajan (2025)^54^	Propofol	100 mg kg^−1^	i.p.	N/A	Mouse	C57BL/6JN	3–4 mo	Male and female	24 h, 72 h, 5 d, 7 d	NR	Increased at 24 h, 72 h, and 5 d but not at 7 d	Unchanged at all timepoints
		75 mg kg^−1^	i.p.	N/A	Mouse	C57BL/6JN	21–24 mo	Male and female	5 d	NR	Increased	Unchanged
		75 mg kg^−1^ every fifth day	i.p.	21 days	Mouse	C57BL/6JN	3–4 mo	Male and female	21 d	NR	Increased	Unchanged
Li (2015)^55^	Propofol	25 mg kg^−1^	i.p.	N/A	Mouse	C57BL/6	15–20 d	NR	5 min	NR	β3 subunit cell-surface expression increased	NR
Zurek (2014)^34^	Isoflurane	0.7 vol%	INH	20 min	Mouse	C57BL/6J×SvEv129	1–4 mo	Male	24 h	NR	Unchanged	Unchanged
		1.3 vol%	INH	1 h	Mouse	C57BL/6J×SvEv129	1–4 mo	Male	24 h	NR	Increased	Unchanged
Zhao (2019)^56^	Isoflurane	1.3 vol%	INH	1 h	Rat	Wistar	3, 24 mo	Male	24, 72 h	Increased at 24 h but not 72 h in 3-month-old rats. Decreased at 24 and 72 h in 24-month-old rats	NR	NR
Zhang (2020)^15^	Isoflurane	1.3 vol%	INH	NR	Mice	C57BL/6J	16 mo	Female	1, 3, 7, 10 d	Unchanged at all timepoints	Unchanged at unspecified timepoint	NR
Gao (2019)^63^	Isoflurane	1.33 vol%	INH	1 h	Mice	C57BL/6J	3–5, 18–20 mo	Male	24 h	NR	NR	Unchanged in both age groups
Wang (2024)^58^	Sevoflurane	4 vol%	INH	2 h	Mice	C57BL/6J	2–3, 18–19 mo	Female	24 h, 72 h, 7 d	NR	Unchanged at all timepoints in 2–3-mo-old mice. Increased at all timepoints in 18–19-mo-old mice	Unchanged at all timepoints in 2–3-mo-old mice. Increased at 72 h but not 24 h or 7 d in 18–19-mo-old mice
Zhang (2025)^59^	Sevoflurane	3.3 vol%	INH	2 h	Mice	C57BL/6	18 mo	Male	24 h	NR	Unchanged	NR
Wang (2024)^62^	Sevoflurane	2.7–3.0 vol%	INH	1 h	Mice	C57BL/6	3 mo	Female	24 h	NR	NR	Unchanged

Neither isoflurane nor sevoflurane typically alters total ɑ5GABA_A_ receptor protein levels,^34^^,^^58^^,^^62^^,^^63^ indicating that increases in cell-surface expression of ɑ5GABA_A_ receptors likely result from increased exocytosis or reduced endocytosis rather than the synthesis of new receptors. Interestingly, the effects of isoflurane on ɑ5 subunit mRNA levels are inconsistent (Table 1), suggesting varying transcriptional responses.^15^^,^^56^ Notably, low-dose isoflurane reduces ɑ5 subunit mRNA expression in aged rats but increases mRNA levels in young rats, further highlighting the potential for complex, age-dependent changes.^56^

### Ketamine and nitrous oxide

Only a limited number of studies have explored the acute and sustained effects of ketamine and nitrous oxide on GABA_A_ receptor function and cell-surface expression. Ketamine directly and rapidly potentiates the function of extrasynaptic GABA_A_ receptors but not synaptic GABA_A_ receptors *in vitro*.^31^ However, under baseline conditions, unlike most GABAergic anaesthetic drugs, ketamine does not cause sustained changes in ɑ5GABA_A_ receptor cell-surface expression or in the amplitude of the tonic current, at least when measured 24 h after drug treatment.^61^ Similarly, nitrous oxide does not acutely change the cell-surface expression of ɑ5GABA_A_ receptors nor the amplitude of GABA-evoked currents, when measured 24 h after drug treatment, under baseline conditions.^64^

### Additional perioperative drugs

**Fig 2:**
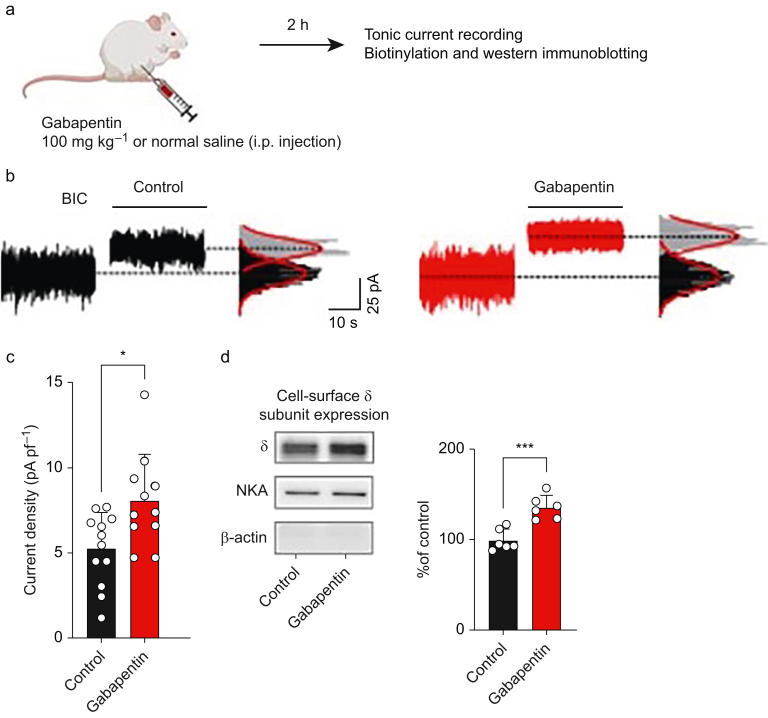
Gabapentin increases δ γ-aminobutyric acid type A (δGABA_A_) receptor cell-surface expression, increases the amplitude of the tonic current recorded in granule cells, and causes neurocognitive deficits. Modified with permission from Yu and colleagues.^13^ (a) A schematic diagram showing the experimental design and timeline. Created in BioRender. (b) Representative traces of whole-cell currents recorded from granule neurones in *ex vivo* cerebellar slices isolated from mice treated with gabapentin or saline. Application of the competitive antagonist bicuculline (BIC) reduces the holding current as evidenced by the upward shift from the baseline. The reduction in current amplitude (i.e. size of the shift from baseline) is greater in neurones from gabapentin-treated mice. The all-points histograms, shown to the right of the current traces, illustrate the shift from baseline current. (c) Quantified data showing the shift from baseline caused by BIC in neurones from controls and gabapentin-treated mice. (d) Representative western blots and summarised data of cell-surface expression of δ subunits relative to the housekeeping proteins Na/K-ATPase (NKA) and β-actin. Data are presented as mean (sem). ∗*P*<0.05, ∗∗∗*P*<0.001.

Other medications administered to surgical patients may alter the cell-surface expression of extrasynaptic GABA_A_ receptors and thereby impact postoperative cognitive recovery. Gabapentin (typically administered as a component of multimodal analgesia) is of interest owing to its associations with PNDs,^65^ and with sedation, dizziness, and ataxia.^66^ Gabapentin is a synthetic analogue of GABA, but it does not bind directly to GABA_A_ receptors.^67^ We discovered that gabapentin increases the cell-surface expression of δGABA_A_ receptors in multiple brain regions including the cerebellum and hippocampus,^13^ and causes a corresponding increase in the tonic current (Fig. 2).^67^ The effects of gabapentin are subunit selective, as gabapentin does not alter the cell-surface expression of α1 subunit- or α5 subunit-containing GABA_A_ receptors.^13^

Behavioural studies using wild-type and mutant mice lacking the *Gabrd* gene that encodes the δ subunit (*Gabrd-/- mice*) showed that δGABA_A_ receptors contribute to the anxiolytic and ataxic effects of gabapentin, but not anti-nociceptive properties.^13^ Increased δGABA_A_ receptor function is also associated with decreased respiratory drive and upper airway tone.^68^ These results are important as population-level studies of patients who were prescribed opioids have shown a 60% increase in opioid-related deaths among those concurrently taking gabapentin.^69^ It is possible that increased expression of δGABA_A_ receptors contributes to reduced respiratory drive in the presence of opioids. Moreover, the combination of the sedative, ataxic, and respiratory-depressant effects of gabapentin may further contribute to the development of PNDs.

Benzodiazepines are also of interest given their association with delirium in patients treated in ICUs.^70^ Recent work has challenged the notion that benzodiazepines increase the risk of postoperative delirium,^71^^,^^72^ but this question nevertheless remains an area of active discovery. The effects of benzodiazepines on the expression pattern of GABA_A_ receptor subtypes have been comprehensively reviewed.^73^ However, most studies examined the consequences of chronic drug treatment in an effort to understand mechanisms underlying benzodiazepine tolerance and withdrawal. Interestingly, prolonged exposure to a benzodiazepine (days to weeks) typically reduces, rather than increases, cell-surface expression of GABA_A_ receptors.^74^^,^^75^

Several studies have examined the effect of benzodiazepines on α5 subunit mRNA and extrasynaptic GABA_A_ receptors. A decrease in α5 subunit mRNA in whole-brain preparations was observed in rats treated with flurazepam (40 mg kg^−1^ i.p.). This reduction was detectable 2 h after drug treatment and levels continued to decline after daily injections for 2–4 weeks.^76^ Another study observed a decrease in α5 subunit mRNA levels in the cortex and hippocampus after 2 weeks of flurazepam treatment (100–150 mg kg^−1^ p.o.); however, levels normalised after 4 weeks of treatment.^77^ Similarly, two studies reported decreases in α5 subunit mRNA levels in the hippocampus and cortex after treatment with diazepam for 3 weeks in rats^78^ and after 1 week of treatment in mice.^79^ In contrast to these results, several studies reported no change in α5 subunit mRNA in the hippocampus or cortex of rats after chronic treatment with flurazepam^80^^,^^81^ or in mice chronically treated with diazepam.^82^ Other studies observed an increase in α5 subunit mRNA in the cortex of rats chronically treated with diazepam.^83^, ^84^, ^85^, ^86^ Interestingly, one study demonstrated increased expression of α5 subunit protein levels after chronic treatment with diazepam.^87^ Also, a study that used quantitative receptor autoradiography (using [^3^H]RY-80 to radiolabel ɑ5GABA_A_ receptors) reported that chronic administration of either flurazepam or diazepam decreased radioligand binding (reflecting decreased receptor expression) in rat hippocampus for several days.^88^

The effect of benzodiazepines on expression of the δ subunit has not been extensively studied. However, chronic treatment with diazepam increases α4 subunit mRNA levels (α4 subunits typically bind with δ subunits) but decreases γ2 subunit mRNA,^83^ suggesting increases in δ subunit expression.

Overall, the effects of benzodiazepines on extrasynaptic GABA_A_ receptor expression and function in the hippocampus and the cortex vary depending on the experimental paradigm and the duration of drug treatment.^80^^,^^87^^,^^89^ The results suggest that any contribution of benzodiazepines to delirium may be through mechanisms that are independent of excess cell-surface expression of GABA_A_ receptors. The potential sustained effects of benzodiazepines on GABA_A_ receptor expression remain an area of active study, given the growing clinical interest in the short-acting benzodiazepine remimazolam as a cognitive-sparing sedative drug.^90^ It is unknown whether remimazolam alters the cell-surface expression of GABA_A_ receptors.

## Proposed mechanisms underlying γ-aminobutyric acid type A receptor overactivity after anaesthesia and surgery

### Astrocyte-neurone crosstalk mediates excess cell-surface ɑ5 γ-aminobutyric acid type A receptors

The cellular and molecular mechanisms underlying the anaesthetic-induced increase in cell-surface expression of ɑ5GABA_A_ receptors have been characterised using primary cultures of neurones and astrocyte-neurone cocultures. These studies have shown that the increase in cell-surface expression of ɑ5GABA_A_ receptors in neurones may not result from direct actions on the neurones themselves, as treating cultured neurones (or neurones grown in cocultures with microglia) with GABAergic anaesthetic drugs does not increase their cell-surface expression as reported *in vivo*.^91^ In contrast, treating astrocyte-neurone cocultures with GABAergic anaesthetic drugs reproduces the *in vivo* finding of sustained upregulation of ɑ5GABA_A_ receptors on the surface of neurones.^91^

Astrocytes, which are the most abundant glial cells, express anaesthetic-sensitive GABA_A_ receptors.^92^ It was found that general anaesthetic drugs activate astrocytic GABA_A_ receptors (likely α4 subunit-containing GABA_A_ receptors) and the resulting efflux of anions causes depolarisation and an influx of calcium, leading to the release of paracrine factors that have yet to be identified.^91^ These soluble factors, which are released from astrocytes, act on interleukin-1β (IL-1β)-dependent signalling pathways in neurones that in turn increase the cell-surface expression of ɑ5GABA_A_ receptors.^14^ Notably, blocking IL-1 receptors or inhibiting downstream signalling factors, including p38 mitogen-activated protein kinase (MAPK), prevents excess cell-surface expression and function of ɑ5GABA_A_ receptors.^14^^,^^91^ Strategies that prevent the release of paracrine factors from astrocytes or block the downstream signalling cascades activated by these factors may represent unique avenues to mitigate PNDs.

Interestingly, the α4 subunit-containing GABA_A_ receptors that are predominantly expressed by astrocytes lack a benzodiazepine binding site.^93^ This further supports the notion that mechanisms other than increases in GABA_A_ receptor cell-surface expression contribute to any sustained cognitive effects of benzodiazepines.

### Inflammation and surgery enhance extrasynaptic γ-aminobutyric acid type A receptor activity

Inflammation caused by aseptic surgical trauma, comorbidities, underlying diseases that necessitate surgery, or a combination of these factors is increasingly recognised as a major contributor to PNDs.^94^^,^^95^ Elevated levels of pro-inflammatory and neuronal injury biomarkers can be readily identified in serum and cerebrospinal fluid from post surgical patients,^96^ and increased levels of inflammatory factors correlate with the risk of developing PNDs.^97^^,^^98^ Results from preclinical models are consistent with these clinical findings, as animals undergoing laparotomy show increased expression of pro-inflammatory interleukins, particularly IL-1β, IL-6, and IL-8, and tumour necrosis factor (TNF) across multiple brain regions.^99^^,^^100^ Interestingly, molecular changes, and corresponding deficits in performance of postoperative cognitive behavioural tasks, are more pronounced in animals that undergo anaesthesia and surgery than in animals exposed to anaesthesia alone.^99^ Cognitive deficits after surgery have been consistently replicated across several rodent models.^15^^,^^99^, ^100^, ^101^, ^102^, ^103^, ^104^, ^105^, ^106^, ^107^, ^108^, ^109^, ^110^ Notably, inflammatory changes and cognitive deficits also occur after major surgery performed under local anaesthesia rather than general anaesthesia in a mouse model.^100^ Thus, both anaesthesia and surgery alone are sufficient rather than necessary to produce persistent cognitive deficits in animal models.

Cell-surface expression of ɑ5GABA_A_ receptors appears to be particularly sensitive to pro-inflammatory signalling. It was observed that IL-1β increases cell-surface expression of ɑ5GABA_A_ receptors, enhances the amplitude of the tonic current, suppresses LTP, and impairs memory.^14^ Pharmacological blockade or genetic deletion of ɑ5GABA_A_ receptors prevents IL-1β-induced cognitive deficits.^14^ Similarly, inhibition or deletion of IL-1β or TNF-ɑ abolishes postoperative memory deficits in animal models.^108^^,^^111^ Consistent with these findings, the combination of anaesthesia and laparotomy increases cell-surface expression of ɑ5GABA_A_ receptors and cognitive deficits to a greater degree than after anaesthesia alone.^15^^,^^104^

Increases in ɑ5GABA_A_ receptor function contrast with the changes in other GABA_A_ receptor populations. For example, chronic inflammation typically reduces the frequency of spontaneous miniature inhibitory postsynaptic currents,^112^ reflecting either decreased GABA release from presynaptic terminals or a loss of inhibitory synapses. Both changes have been observed in models of inflammation^112^, ^113^, ^114^, ^115^ and surgery.^15^^,^^116^

Both IL-1β and GABAergic anaesthetic drugs trigger cytosolic signalling cascades that converge on at least one similar component: MAPK.^91^^,^^117^^,^^118^ Studies are needed to determine whether inflammation and anaesthetic drugs have synergistic or parallel effects on GABA_A_ receptor function and, hence, postoperative cognitive function.

## Prevention and treatment of perioperative neurocognitive disorders

The evidence presented above suggests that reducing excess cell-surface expression of extrasynaptic GABA_A_ receptors is a plausible strategy to mitigate PNDs. Pharmacologically targeting α5GABA_A_ receptors is attractive for several reasons. Firstly, the expression of these receptors is restricted to certain types of neurones and several distinct brain regions that regulate cognitive processes.^35^^,^^36^ Secondly, pharmacological inhibitors that allosterically reduce the function of α5GABA_A_ receptors exhibit good target engagement in the brain and lack the adverse side-effects of non-selective antagonists of GABA_A_ receptors including pro-convulsant and anxiogenic effects.^119^ Thirdly, radixin (the anchoring protein that tethers extrasynaptic α5GABA_A_ receptors to the actin cytoskeleton and regulates cell-surface localisation and stability) has unique affinity for this receptor subpopulation. Thus, disrupting the radixin-α5GABA_A_ receptor complex is a selective and plausible target for therapeutic intervention.^120^^,^^121^

The most practical approach to reducing α5GABA_A_ receptor cell-surface expression is repurposing drugs with neuroprotective properties that have already received regulatory approval. Selective allosteric inhibitors of ɑ5GABA_A_ receptors, which were developed for other clinical indications, may also be beneficial, but these drugs require further study. In addition, we have proposed the potential utility of a novel decoy peptide that disrupts radixin-α5GABA_A_ receptor interaction and prevents the anchoring of α5GABA_A_ receptors to the cytoskeleton, thereby reducing extrasynaptic localisation.^122^^,^^123^ Finally, we advise against the use of gabapentin in patients at high risk of developing PNDs. Each of these strategies shows some promise for restoring cognitive function, as described below.

### Dexmedetomidine

Dexmedetomidine is an ɑ_2_-adrenergic receptor agonist with sedative, analgesic, and sympatholytic properties.^124^ It reduces the incidence and severity of delirium among patients admitted to the ICU, compared with other sedative agents.^125^^,^^126^ The evidence to date for the use of dexmedetomidine in surgical patients is less compelling.^127^, ^128^, ^129^, ^130^, ^131^ Nevertheless, a 2025 Practice Advisory from the ASA endorsed dexmedetomidine as the only drug supported by clinical evidence for consideration among older adults to prevent postoperative delirium.^132^

Dexmedetomidine, when administered during surgery, may preserve cognition by several mechanisms. It reduces the required dose of GABAergic anaesthetic drugs^133^ and exhibits anti-inflammatory properties.^134^^,^^135^ Preclinical studies have identified a third possible mechanism: dexmedetomidine prevents etomidate- and sevoflurane-induced excess cell-surface expression of α5GABA_A_ receptors.^49^ Consistent with these *in vitro* results, dexmedetomidine prevents memory and problem-solving deficits after etomidate and sevoflurane administration in young adult mice (without any adjustment of etomidate or sevoflurane dose).^49^

**Fig 3:**
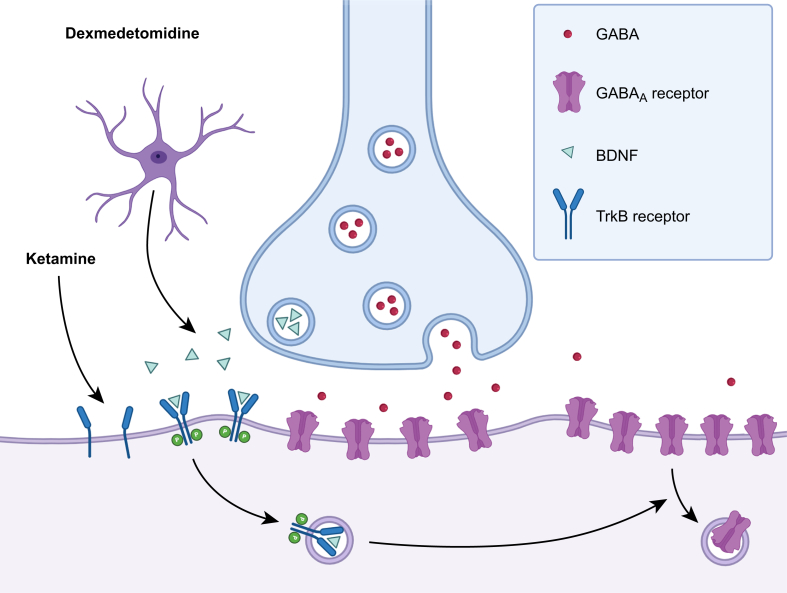
Cotreatment with dexmedetomidine and ketamine reduces the anaesthetic-induced increase in ɑ5 γ-aminobutyric acid type A (ɑ5GABA_A_) receptor expression and function. Dexmedetomidine activates α_2_-adrenergic receptors in astrocytes, stimulating the release of brain-derived neurotrophic factor (BDNF), which binds to tropomysin receptor kinase B (TrkB) receptors expressed on the surface of neurones. BDNF binding promotes receptor dimerisation and internalisation, which activates signalling cascades that reduce excess ɑ5GABA_A_ receptor cell-surface expression after general anaesthesia. In contrast, ketamine increases TrkB receptor cell-surface expression in neurones (rather than increasing BDNF levels) and reduces ɑ5GABA_A_ receptor cell-surface expression after general anaesthesia. Created in BioRender.

Dexmedetomidine is thought to protect neurones by activating α_2_ adrenergic receptors in astrocytes, which triggers the release of neurotrophic factors.^49^ Specifically, dexmedetomidine stimulates astrocytes to release brain-derived neurotrophic factor (BDNF), a molecule that regulates cognitive function. BDNF acts on tropomyosin receptor kinase B (TrkB) receptors in neurones, ultimately reducing cell-surface expression of α5GABA_A_ receptors (Fig. 3).^49^ In surgical patients, dexmedetomidine increases levels of BDNF^136^ and reduces neuroinflammation.^137^ Collectively, these mechanisms may contribute to the effectiveness of dexmedetomidine at preventing PNDs.

### Ketamine

In clinical trials, the cognitive-sparing properties of ketamine are less convincing than those of dexmedetomidine. Clinical studies that examined the relationship between perioperative treatment with ketamine and cognitive performance after surgery were recently reviewed.^138^ Approximately one-third of the 58 clinical trials that met the criteria for inclusion in the review reported a reduced incidence or duration of PNDs among patients treated with ketamine,^138^ suggesting that some groups of patients may benefit. As with dexmedetomidine, it remains unclear whether ketamine preserves cognition in patients by allowing reduced doses of other anaesthetic drugs that target GABA_A_ receptors, by reducing inflammation, or by engaging other neuroprotective mechanisms.^138^ However, ketamine also appears to reduce neuroinflammation^139^ and increase BDNF levels, suggesting potential effects on α5GABA_A_ receptors.^140^

In animal models, ketamine alone has no effect on baseline levels of α5GABA_A_ receptors, the amplitude of the tonic current, or memory performance; however, ketamine prevents excess cell-surface expression of ɑ5GABA_A_ receptors caused by GABAergic anaesthetic drugs.^61^ Specifically, ketamine (10 μM) prevents etomidate-induced (1 μM) and sevoflurane-induced (266 μM) excess ɑ5GABA_A_ receptor cell-surface expression, and prevents the corresponding increase in tonic current in hippocampal neurones measured 24 h after drug co-treatment.^61^ Complementary behavioural studies showed that a subanaesthetic dose of ketamine (10 mg kg^−1^ i.p.) prevents sevoflurane-induced (2.3 vol%, 2 h) deficits in recognition and spatial memory, when measured 24 h and 48 h after drug treatment, respectively.^61^

Like dexmedetomidine, the neuroprotective effect of ketamine on excess cell-surface expression of ɑ5GABA_A_ receptors depends on the BDNF-TrkB signalling pathway.^61^ Whereas ketamine was found to not change BDNF levels under the experimental conditions, it did increase cell-surface expression of TrkB receptors,^61^ which would be expected to enhance BDNF-TrkB signalling (Fig. 3). Interestingly, chronic exposure to ketamine (30 mg kg^−1^ i.p. daily for 1 or 3 months) in mice, which may mimic ketamine substance use disorder in humans, upregulates ɑ5GABA_A_ receptor mRNA and protein levels in the prefrontal cortex, which is associated with working memory deficits.^141^ Thus, ketamine’s effects on cognition appear to be dependent on cumulative dose.

### ɑ5 γ-Aminobutyric acid type A receptor inhibitors

**Fig 4:**
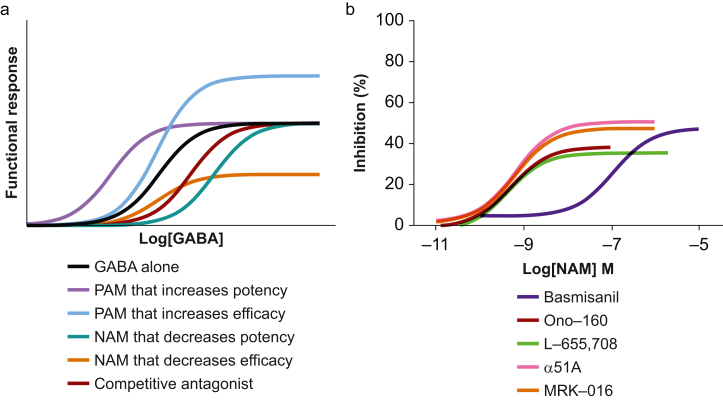
Conceptual and experimental responses of γ-aminobutyric acid type A (GABA_A_) receptors in the absence and presence of various pharmacological modulators. (a) GABA_A_ receptor concentration-response plots illustrate the effects of an orthosteric agonist (GABA) and various drugs from different classes of modulators. The functional response to GABA is shown in black. Allosteric drugs bind at sites that are distinct from the orthosteric site and indirectly influence receptor function. Negative allosteric modulators (NAMs), also known as inverse agonists, decrease agonist potency and shift the curve to the right. Some NAMs also lower agonist efficacy, reducing the maximal response. Positive allosteric modulators (PAMs) have the opposite effects and shift the response curve to the left. PAMs can also increase the maximal efficacy of the agonist. A competitive antagonist occupies the same binding site as the orthosteric agonist, and competes with the natural ligand, causing a rightward shift in the curve. Higher concentrations of the orthosteric agonist can overcome the binding of competitive antagonists. (b) Summarised data illustrating the inhibitory effects of five different NAMs on whole-cell currents evoked by a low concentration of GABA in cultured hippocampal neurones. All the drugs caused a concentration-dependent reduction in the amplitude of the tonic current. The relative potency and efficacy of the α5-specific NAMs are shown. Notably, basmisanil, the most widely studied α5-specific NAM in clinical trials, exhibits the lowest potency. Data are reproduced with permission from Manzo and colleagues.^145^

Two classes of drugs that directly and selectively inhibit ɑ5GABA_A_ receptor function include NAMs and competitive antagonists. A NAM molecule binds to an allosteric site that is distinct from the agonist recognition domain.^142^ The drug induces conformational changes that reduce receptor responsiveness to endogenous ligands (e.g. GABA) by either lowering agonist affinity or reducing downstream responsiveness to the agonist.^142^ NAMs have opposite actions to PAMs (e.g. midazolam), as they shift the GABA concentration-response to the right or reduce the maximum response.^143^ NAMs generally require agonist binding and exhibit a ceiling effect.^144^ In contrast, competitive antagonists bind to the agonist recognition site but they do not produce an inherent biological effect.^144^ Instead, they prevent agonist binding, and increasing the concentration of agonist can overcome their effect. The effects of these various classes of drugs on the concentration-response to GABA are illustrated in Figure 4a.

Multiple pro-cognitive ɑ5-specific NAMs have been developed,^16^ including L-655,708, a5IA (LS-193,268), and basmisanil (RG-1662; RO5186582). These drugs reduce the amplitude of the tonic current,^25^ enhance synaptic plasticity,^146^^,^^147^ and improve cognitive performance in preclinical models.^14^^,^^34^^,^^146^^,^^147^ To date, two studies have showed that L-655,708 improves cognition after anaesthesia and surgery in mouse models.^15^^,^^104^ L-655,708 also prevents anaesthetic-induced memory loss in mice.^12^^,^^34^^,^^57^^,^^58^ Clinical trials of NAMs in surgical patients have not been undertaken; however, a5IA improves alcohol-induced cognitive impairment in healthy volunteers.^148^

Basmisanil, the most widely studied ɑ5-specific NAM, has been examined as a treatment for cognitive impairments associated with Down syndrome^149^ and schizophrenia,^150^ although these trials have not reported benefit in humans. Additional trials of basmisanil for the treatment of cognitive deficits associated with ischaemic stroke^151^ and childhood Dup15q syndrome^152^ were terminated prematurely. The lack of efficacy of ɑ5-specific NAMs may be attributed to the complexity of the pathophysiology underlying the cognitive disorders selected for these clinical trials. PNDs, which have a clearer starting point, may be associated with fewer chronic changes in the central nervous system, and may be more suitable indications for drug trials of ɑ5-specific NAMs. Alternatively, low drug efficacy for reducing the activity of native ɑ5GABA_A_ receptors in neurones may account for the failed clinical trials. Existing ɑ5-specific NAMs have limited and variable efficacies for reducing the amplitude of the tonic current recorded in hippocampal neurones (Fig. 4b).^145^

The competitive ɑ5GABA_A_ receptor antagonist, S44819, also reduces the amplitude of the tonic current, enhances LTP, and improves short and long-term memory in murine models.^153^^,^^154^ Clinical studies revealed that S44819 produces electroencephalographic changes that are consistent with a reduction in tonic current.^155^ To date, S44819 has also been found to be ineffective in treating cognitive deficits in patients after ischaemic stroke, and unlike some ɑ5-specific NAMs, the drug had an excellent safety profile.^155^ It will be important to determine whether S44819 or other competitive antagonists improve cognition after surgery in animal models.

### Targeting ɑ5 γ-aminobutyric acid type A receptor anchoring proteins

**Fig 5:**
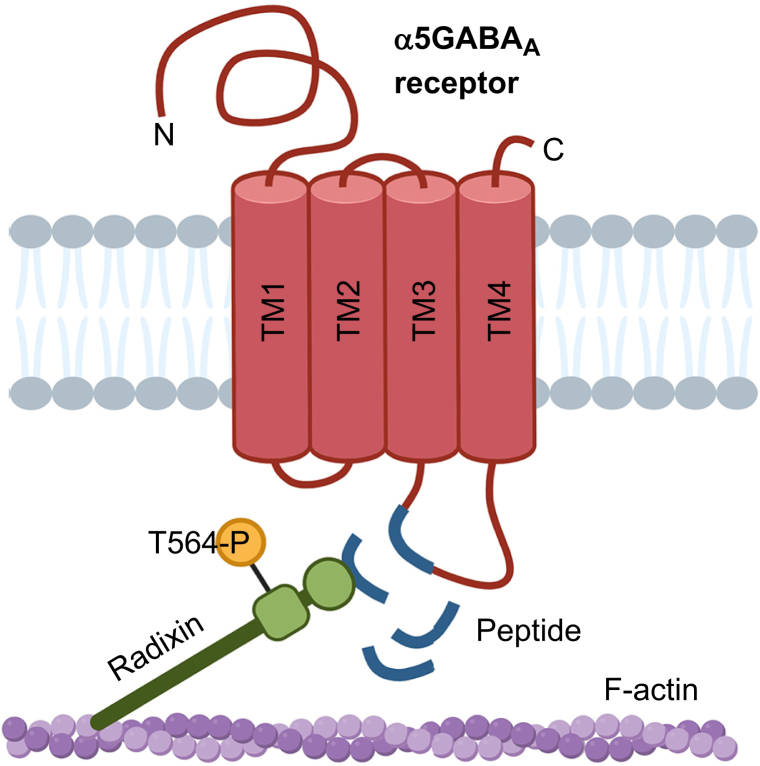
Mechanism of the peptide-mediated disruption of ɑ5 γ-aminobutyric acid type A (ɑ5GABA_A_) receptor cytoskeleton anchoring to radixin. The synthetic sequence-specific peptide mimics the radixin-specific binding motif of the α5 subunit of the GABA_A_ receptors complex and competitively disrupts the interaction between ɑ5GABA_A_ receptors and its main extrasynaptic scaffolding protein, radixin. Radixin phosphorylation at residue T564 induces a conformational change which allows the anchor to bind to the F-actin cytoskeleton. Binding of the peptide destabilises receptors from radixin and thereby reduces the tonic current. Created in BioRender. TM, transmembrane domain.

An alternative approach to reducing extrasynaptic ɑ5GABA_A_ receptor function is to disrupt the binding of receptors to their main extrasynaptic scaffolding protein, radixin. Radixin links ɑ5GABA_A_ receptors to the actin cytoskeleton and prevents diffusion to synapses.^16^^,^^120^^,^^121^ A sequence-specific decoy peptide was developed that selectively targets the radixin-ɑ5GABA_A_ receptor interaction site. The peptide was designed to mimic the essential binding motif on ɑ5GABA_A_ receptors, which is recognised by radixin (Fig. 5). This peptide may reduce the tonic current and mitigate cognitive deficits associated with excess ɑ5GABA_A_ receptor function, as outlined in several patents.^122^^,^^123^ Preclinical studies are underway in our laboratory to determine the effectiveness of the decoy peptide for preventing postoperative cognitive deficits. Similar blocking peptide strategies have proved to be effective in other neurological and non-neurological disorders.^156^

### Perioperative gabapentinoids

As described above, gabapentin causes excess cell-surface accumulation of δGABA_A_ receptors,^13^ consistent with the ‘excess cell-surface GABA_A_ receptor’ hypothesis of PNDs. It will be important to determine whether other members of the gabapentinoid class of drugs, including pregabalin, cause similar effects. Like gabapentin, pregabalin causes sedation, respiratory depression, and memory loss in patients,^157^^,^^158^ suggesting that these effects are also mediated by δGABA_A_ receptors. Overall, gabapentinoids should be used cautiously in patients at risk for PNDs.

## Limitations and future directions

The proposed ‘excess cell-surface GABA_A_ receptor’ hypothesis of PNDs has several important limitations. Preclinical studies have shown that the excess extrasynaptic GABA_A_ receptor activity and cognitive deficits after general anaesthesia vary depending on the specific anaesthetic drug and its dose, the age of the experimental animal, and the methods used to assess receptor activity and cognitive behaviours.^15^^,^^34^^,^^58^^,^^159^ Also, to date, only a limited number of studies have shown that inhibitors of ɑ5GABA_A_ receptors improve cognition after laparotomy in mice,^15^^,^^104^ although several additional studies demonstrate a rescue effect of NAMs after anaesthesia alone. Interestingly, several studies have showed that PAMs that target α5GABA_A_ receptors may improve cognitive deficits associated with ageing, depression, schizophrenia, and neurodevelopmental disorders, which is inconsistent with our hypothesis.^16^ Under certain experimental conditions, PAMs may preferentially act on GABA_A_ receptors expressed in interneurones (e.g. somatostatin-expressing interneurones), rather than excitatory neurones, and thereby improve cognition through alternative pathways.^160^ Overall, it remains unclear how both α5 subunit-specific NAMs and PAMs elicit beneficial cognitive effects in different behavioural paradigms.

Several key clinical translational investigations should be prioritised in future work. In terms of repurposing available neuroprotective drugs, the optimum dose and timing of drug administration of dexmedetomidine and ketamine to prevent PNDs need to be determined. Moreover, it is unknown whether combinations of these drugs produce better cognitive outcomes than either drug alone and whether the cognitive sparing effects of dexmedetomidine and ketamine are modified by co-treatment with other anaesthetic drugs. Also, it will be important to confirm whether dysregulation of BDNF-TrkB signalling pathways contributes to cognitive deficits after surgery in patients.

The development of imaging biomarkers to correlate α5GABA_A_ receptor expression with PNDs is of interest. Positron emission tomography imaging studies to assess α5GABA_A_ receptor expression with ɑ5GABA_A_ receptor-selective markers (e.g. [^11^C]Ro15–4513)^38^ after surgery are plausible, and may provide a biomarker that distinguishes ɑ5GABA_A_ receptor-associated PNDs from other causes of cognitive impairment. Clinical trials of α5GABA_A_ receptor inhibitors including S44819 are also of considerable interest given their success in preclinical models.^154^ Finally, even large-scale clinical trials struggle to compare the cognitive effects of different anaesthetic drugs owing to the multiple patient- and perioperative-specific confounders. International academic consortiums that use real-world observational data collected in large electronic medical records datasets, such as the Multicenter Perioperative Outcomes Group, are needed to inform us about cognitive outcomes after certain combinations of anaesthetic drugs and the potential effectiveness of dexmedetomidine and ketamine as neuroprotectants.^161^

In conclusion, the role of ɑ5GABA_A_ and δGABA_A_ receptors in mediating PNDs has garnered increasing attention, given the mounting evidence that shows general anaesthetic drugs, inflammation, and gabapentin increase extrasynaptic GABA_A_ receptor expression and function. This review summarises alterations in extrasynaptic GABA_A_ receptors after anaesthesia and surgery and describes emerging therapeutic and diagnostic innovations. PNDs remain one of the most common and devastating postoperative complications and there is an urgent need for targeted therapeutic approaches. As we untangle the molecular intricacies of extrasynaptic GABA_A_ receptors, numerous diagnostic tools and approaches for mitigating PNDs lie just beyond the horizon.

## Authors’ contributions

Conceptualisation: JB, CTAB

Literature review: JB, CTAB

Writing—original draft preparation, visualisation: JB, CTAB

Writing—review and editing: all authors

Supervision: BAO

## Funding

University of Toronto Temerty Faculty of Medicine (Comprehensive Research Experience for Medical Students Summer Research Program award to JB); University of Toronto Department of Anesthesiology & Pain Medicine (Dr. Alan K. Laws Clinician Scientist Fellowship Fund and Dr. Alan W. Conn Graduate Award to CTAB); Canadian Anesthesia Research Foundation (Residents' Research Grant to CTAB); Sunnybrook Health Sciences Centre Department of Anesthesiology and Pain Medicine (Dr. Kirk Weber Award to CTAB); Canadian Institutes of Health Research (Foundation Grant to BAO and Vanier Canada Graduate Scholarship to CTAB).

## Declarations of interest

BAO is co-director of the Perioperative Brain Health Centre (Toronto, ON, Canada; www.perioperativebrainhealth.com). She is a named inventor on a Canadian patent (2,852,978) and three US patents (9,517,265, 10,981,954, and 12,054,562). The patents are held by the University of Toronto and are for new methods to prevent and treat delirium and persistent neurocognitive deficits after anaesthesia and surgery, and to treat mood disorders. The other authors declare that they have no conflicts of interest.
